# Anti-Her-2/neu antibody induces apoptosis in Her-2/neu overexpressing breast cancer cells independently from p53 status

**DOI:** 10.1054/bjoc.2001.2197

**Published:** 2001-11

**Authors:** T Brodowicz, D Kandioler, S Tomek, C Ludwig, M Rudas, R Kunstfeld, W Koestler, M Hejna, A Budinsky, C Wiltschke, C C Zielinski

**Affiliations:** Clinical Division of Oncology, Experimental Oncology, Department of Medicine I, Clinical Division of General Surgery, Department of Surgery, Clinical Division of Clinical Pathology, Department of Pathology, University Hospital; , Ludwig Boltzmann Institute for Clinical Experimental Oncology, Vienna, Austria

**Keywords:** apoptosis, Bcl-2, breast cancer, HER-2/neu, p53

## Abstract

Anti-Her-2/neu antibody is known to induce apoptosis in HER-2/neu overexpressing breast cancer cells. However, exact regulatory mechanisms mediating and controlling this phenomenon are still unknown. In the present study, we have investigated the effect of anti-Her-2/neu antibody on apoptosis of HER-2/neu overexpressing human breast cancer cell lines SK-BR-3, HTB-24, HTB-25, HTB-27, HTB-128, HTB-130 and HTB-131 in relation to p53 genotype and bcl-2 status. SK-BR-3, HTB-24, HTB-128 and HTB-130 cells exhibited mutant p53, whereas wild type p53 was found in HTB-25, HTB-27 and HTB-131 cells. All seven cell lines weakly expressed bcl-2 protein (10–20%). Anti-Her-2/neu antibody, irrespective of p53 and bcl-2 status, induced apoptosis in all 7 cell lines dose- and time-dependently and correlated with Her-2/neu overexpression. In addition, incubation of cell lines with anti-Her-2/neu antibody did not alter p53 or bcl-2 expression. Anti-HER-2/neu antibody did not induce apoptosis in HER-2/neu negative HBL-100 and HTB-132 cell lines. Our results indicate that within the panel of tested breast cancer cell lines, anti-Her-2/neu antibody-induced apoptosis was independent from the presence of intact p53. © 2001 Cancer Research Compaign http://www.bjcancer.com


*British Journal of Cancer* (2001) **85**(11), 1764–1770
© 2001 Cancer Research Campaign
doi: 10.1054/ bjoc.2001.2197, available online at http://www.idealibrary.com on IDEAL

### Anti-Her-2/neu antibody induces apoptosis in Her-2/neu overexpressing breast cancer cells independently from p53 status

**T Brodowicz[1], D Kandioler[3], S Tomek[1], C Ludwig[3], M Rudas[4], R Kunstfeld[1], W Koestler[1], M Hejna[1], A Budinsky[1], M Krainer[1], C Wiltschke[1] and CC Zielinski[1,2,5]**

[1]Clinical Division of Oncology; [2]Chair of Medical Experimental Oncology, Department of Medicine I; [3]Clinical Division of General Surgery, Department of Surgery; [4]Clinical Division of Clinical Pathology, Department of Pathology, University Hospital; [5]Ludwig Boltzmann Institute for Clinical Experimental Oncology, Vienna, Austria

**Summary** Anti-Her-2/neu antibody is known to induce apoptosis in HER-2/neu overexpressing breast cancer cells. However, exact regulatory mechanisms mediating and controlling this phenomenon are still unknown. In the present study, we have investigated the effect of anti-Her-2/neu antibody on apoptosis of HER-2/neu overexpressing human breast cancer cell lines SK-BR-3, HTB-24, HTB-25, HTB-27, HTB-128, HTB-130 and HTB-131 in relation to p53 genotype and bcl-2 status. SK-BR-3, HTB-24, HTB-128 and HTB-130 cells exhibited mutant p53, whereas wild type p53 was found in HTB-25, HTB-27 and HTB-131 cells. All seven cell lines weakly expressed bcl-2 protein (10–20%). Anti-Her-2/neu antibody, irrespective of p53 and bcl-2 status, induced apoptosis in all 7 cell lines dose- and time-dependently and correlated with Her-2/neu overexpression. In addition, incubation of cell lines with anti-Her-2/neu antibody did not alter p53 or bcl-2 expression. Anti-HER-2/neu antibody did not induce apoptosis in HER-2/neu negative HBL-100 and HTB-132 cell lines. Our results indicate that within the panel of tested breast cancer cell lines, anti-Her-2/neu antibody-induced apoptosis was independent from the presence of intact p53. © 2001 Cancer Research Compaign

**Keywords**: apoptosis; Bcl-2; breast cancer; HER-2/neu; p53

**Abbreviations**: FCS, fetal calf serum; FITC-dUTP, fluorescent tagged deoxyuridine triphosphate; TdT, terminal deoxynucleotidyl transferase; PI, propidium iodide

The Her-2/neu gene is a proto-oncogene from the *erb*B family of receptor tyrosine kinases, located on chromosome 17q21 (Coussens et al, 1985) and encodes for a 185 KD transmembrane glycoprotein containing an extracellular domain and intracellular tyrosine kinase activity. While the extracellular domain possesses ligand-binding activity (Lupu et al, 1990), the direct natural ligand of Her-2/neu is still unknown. HER-2/neu gene amplification or protein overexpression was reported in various types of malignancies including ovarian, gastric, lung cancer and in 10 to 40% of primary human breast cancers as well as in several human breast carcinoma cells (Kraus et al, 1987).

Impressive results of the combination of anti-Her-2/neu antibody termed trastuzumab with cytotoxic drugs in patients with advanced breast cancer overexpressing Her-2/neu (Fornier et al, 1999) have been reported (Slamon et al, 1998, 2001; Norton et al, 1999; Burstein et al, 2001) to result in a significant increase in response rate and duration of time elapsed to disease progression as well as overall survival. However, also the administration of anti-Her-2/neu antibody as a single agent in pretreated patients with Her-2/neu overexpressing tumours was able to produce an objective response rate of 15% of considerable duration (Cobleigh et al, 1998, 1999).

*Received 27 March 2001*
*Revised 2 October 2001*
*Accepted 4 October 2001*

*Correspondence to:* CC Zielinski

In vitro, anti-Her-2/neu antibody-mediated inhibition of proliferation and mediation of lysis by lymphokine-activated killer (LAK) cells has been shown to be also closely related to the overexpression of Her-2/neu in breast cancer cell lines (Harwerth et al, 1992, 1993; Brodowicz et al, 1997). Although the underlying molecular events of these interactions remain unclear, a multitude of possibilities has been discussed (Kerbel, 1999). Apart from the obvious immunologic interaction between Her-2/neu protein and the antibody, anti-Her-2/neu antibody has been hypothesized to also have properties to act as an antiangiogenic agent.

The present investigation focused upon the ability of anti-Her-2/neu antibody to induce apoptosis in Her-2/neu overexpressing breast cancer cells with particular emphasis upon the regulatory molecular requirements for the proper induction of programmed cell death. In response to DNA damage, wild type p53 has been shown to be responsible for the regulation of the cell cycle (Harvey et al, 1993) and to induce either cell cycle arrest in the G1 phase (Di Leonardo et al, 1994; Kastan et al, 1991) allowing for DNA repair or apoptosis (Yonish-Rouach et al, 1991). In addition, several target genes, encoding proteins like insulin-like growth factor-1-binding protein 3 (Buckbinder et al, 1995), Fas/Apo-1/CD95 (Owen-Schaub et al, 1995), KILLER DR5 (Wu et al, 1997), bax (Yin et al, 1997), reactive oxygen radicals (Johnson et al, 1996) or PAG608 (Israeli et al, 1997), might be induced by p53. Subsequently, these proteins can promote apoptosis by affecting receptor signaling or apoptotic effector proteins. However, additional p53-independent apoptotic pathways exist in normal as well as malignant cells which differ in their sensitivity towards apoptosis-inducing agents (Thompson, 1995; Bracey et al, 1995; Shao et al, 1995; Delia et al, 1993). Exact mechanisms involved in p53-independent apoptosis are poorly understood. Loss of retinoblastoma (pRb) family function with subsequent release and deregulation of E2F-1 protein (Dyson, 1998), induction of reactive oxygen radicals (Venot et al, 1998) and suppression of sequence-specific transactivation-mediated growth arrest (Sionov and Haupt, 1999) by a proline-rich domain of the human p53 might contribute to p53-independent apoptosis. Furthermore, direct interaction with apoptosis inducing proteins XPB and XPD (Wang et al, 1996) or proteins, which interact with anti-apoptotic proteins 53BP2 (Naumovski and Cleary, 1996) may represent p53-independent apoptosis inducing mechanisms. Bcl-2 prevents apoptosis by inhibiting reactive oxygen intermediates formation (Hockenberry et al, 1993) as well as mitochondrial apoptosis-inducing factor-(AIF) and cytochrome C release (Dragovich et al, 1998; Susin et al, 1999). Also p53-dependent apoptosis is repressed by bcl-2 (Chiou et al, 1994), partly due to impeding nuclear p53 import (Beham et al, 1997). The apoptosis-regulating aspects of p53 in the context of anti-Her-2/neu antibody were of particular interest, as previous studies have demonstrated that growth arrest and induction of apoptosis resulting from appropiate chemo- and/or radiotherapeutic measures were largely dependent upon the intact function of the p53 gene in various models (Bergh et al, 1995; Elledge et al, 1995; Sarkis et al, 1995), and the clinical observation that patients with malignancies exhibiting high frequency of p53 mutations exhibited resistance to cytotoxic agents (Aas et al, 1996).

As these functional aspects have been insufficiently addressed and not fully elucidated in the functional context of anti-Her-2/neu antibody until now, we have studied the molecular aspects of anti-Her-2/neu antibody-mediated induction of apoptosis in human breast cancer cell lines SK-BR-3, HTB-24, HTB-128 and HTB-130 which exhibited mutant p53 and HTB-25, HTB-27 and HTB-131 with wild type p53.

### MATERIALS AND METHODS

### Cell cultures

Cell lines were obtained from American Type Culture Collection (ATCC). SK-BR-3 (human breast carcinoma) and HBL-100 (human mammary epithelial) were cultured in McCoy's 5A medium supplemented with 10% heat-inactivated fetal calf serum (FCS) (all from GIBCO Life Technologies Ltd, Paisley, Scotland, UK), 50 units/ml penicillin, 50 µg streptomycin and 2 mM L-glutamine (all from HyClone, Europe Ltd, Cramlington, UK)/ml medium. The human breast carcinoma cell lines HTB-24 (MDA-MB-157), HTB-25 (MDA-MB-175-VII), HTB-27 (MDA-MB-361), HTB-128 (MDA-MB-415), HTB-130 (MDA-MB-436), HTB-131 (MDA-MB-453) and HTB-132 (MDA-MB-468) were cultured in Leibovitz's L-15 medium with L-glutamine (PAA Laboratories Gmbh, Linz, Austria) supplemented with 10% heat-inactivated FCS (Gibco), 50 U penicillin and 50 µg streptomycin (all from HyClone) per ml. Cells were grown as monolayers (standard conditions) in T75 flasks (Falcon, Becton Dickinson Comp., NJ, USA) at 37˚C in a humidified atmosphere with free gas exchange without $CO_2$ by seeding $5 \times 10^6$ cells in 25 ml of appropriate medium. SK-BR-3 and HBL-100 cells were cultured in a humidified atmosphere containing 5% $CO_2$.

### ANTI-HER-2/NEU ANTIBODY

A commercially available c-*erb*B-2 monoclonal mouse $IgG_1$ antibody (Clone TAb 250) (Zymed Laboratories Inc., South San Francisco, CA, USA) was used. This antibody immunoprecipitates a protein of 185 KD from a [$^{35}$S] labeled lysate of NIH3T3 cells transfected with the c-*erb*B-2 gene. In addition, this antibody has been shown to recognize the external domain of the c-*erb*B-2 gene product from radiolabeled, permanently transfected CHO cells. Furthermore, the antibody of this clone (TAb 250) exerted a Her-2/neu specific antiproliferative impact on previously tested breast cancer lines (Brodowicz et al, 1997). For assays of proliferation inhibition and apoptosis 25 µg c-*erb*B-2 antibody was dissolved in one ml of distilled water and subsequently diluted in appropriate culture medium (final concentrations: 0.025 µg/ml, 0.25 µg/ml and 2.5 µg/ml). A preparation of mouse IgG1 antibody (Serotec, Oxford, UK) was used for control experiments.

### Detection of Her-2/neu by immunofluorescence

After harvesting and 3 washes with HBSS (Gibco Life Technologies Ltd, Paisley, Scotland, UK) cell lines ($10^6$ cells/sample) were preincubated with 20% human AB-group serum for 20 min at room temperature. Afterwards cells were washed and incubated with 50 µl of appropriately diluted anti-Her-2/neu antibody for 30 min on ice. Isotype matched mouse antibodies were used as controls (Immunotech, Marseille, France). After washing cells were incubated with fluorescein isothiocyanate (FITC) labeled goat anti-mouse IgG antibodies (Immunotech, Marseille, France) for 30 min on ice. Cells were then washed three times and resuspended in 300 µl staining buffer supplemented with 7 amino-actinomycin-D (7-AAD) (final concentration: 1 µg/ml) (Sigma, Steinheim, Germany) to allow exclusion of dead cells. Subsequently, cells were analysed by flow cytometry on a FACScan (Becton Dickinson, CA, USA).

### p53 Sequence analysis

Total genomic tumor DNA was extracted from $2 \times 10^7$ cells using standard phenol–chloroform extraction methods. Exons 2 to 11 of the p53 gene were amplified separately using oligonucleotide primers placed in the adjacent intron regions as described previously (Lehmann et al, 1991; Brodowicz et al, 1999). PCR products were controlled for purity, quantity and quality by subjecting 5 µl of PCR products to pre-cast 6% acrylamide/bis-acrylamide gels (Novex, San Diego, CA, USA) using the pBR322 DNA-Msp I digest as reference standard (Clontech Lab.Inc., Palo Alto, CA, USA). To remove residual single-stranded primers, 5 µl of PCR products were enzymatically treated with combination of exonuclease I and shrimp alkaline phosphatase (United States Biochemical, Cleveland, OH, USA). Pretreated PCR products were then directly sequenced using the Cycle Sequencing Kit (Roche Molecular Systems Inc., Branchburg, NJ, USA), utilizing Ampli Taq$^R$ DNA Polymerase and $\alpha^{35}$ S labeled dATP (DuPont NEN, Brussels, Belgium). For each reaction (for the four nucleotides), 0.5 µl of the thermostable DNA polymerase provided in the kit were added at last to the reaction mix containing 2 µl reaction buffer, 2–4 µl of enzymatically pretreated PCR product, 0.5 pmol unique primer (separate reaction for each exon) and water to adjust total volume to 20 µl. After running, the 6% acrylamide/bis-acrylamide gels are dried for 90 min at 70˚ and directly subjected to autoradiography against the Bio Max-MR film (Kodak, New

*© 2001 Cancer Research Campaign*

Haven, CT, USA). Mutations found were confirmed by at least one complete reanalysis.

### p53 Immunohistochemistry p53 protein staining was performed with a mouse monoclonal IgG2a antibody (Clone: DO-1; Immunotech, Marseille, France; diluted 1:20) directed against wild type and mutant p53 protein. Cytocentrifugates were fixed with Merckofix fixation spray (Merck, Darmstadt, Germany). After blocking with horse serum, samples were incubated with the primary antibody for 1 h. Further immunohistochemical staining was performed according to the ABC-method, using products from Vector Laboratories (Burlingame, CA, USA). Briefly, after incubation with the primary antibody and incubation with a biotinylated secondary-antibody, incubation with the ABC complex for 45 min followed. The reaction product was developed with 3,3′-diaminobenzidine tetrahydrochloride. Finally, slides were counterstained with Gill's haematoxylin. All steps of incubation were performed at room temperature. A cell line was scored negative when nuclear staining was rare (<10%) or absent (Kandioler-Eckersberger et al, 2000).

### Cell proliferation assay [$^3$H]Thymidine incorporation assay

Cells were plated in 96-well microtiter plates (Costar, Cambridge, MA, USA) at a density of $5 \times 10^4$ cells/well. Subsequently, anti-Her-2/neu preparations in varying concentrations were added to cell lines (see above), which had adhered for 1 h, and subsequently cultured for 24, 48, 72 and 96 h at 37˚C in a humidified atmosphere under the appropriate conditions ( = with or without 5% $CO_2$). [$^3$H] Thymidine (Amersham International Life Science, PLC, Buckinghampshire, UK), at a concentration of 0.5 µCi/well, was included for the final 16 h. The incorporation of [$^3$H]Thymidine into DNA was measured by a Direct Beta Counter-Matrix 96 (Packard, Groningen, Netherlands) after the cells were harvested with the Harvester Micromate 196 (Packard, Groningen, Netherlands) onto Glass Fiber Filters (Packard, Groningen, Netherlands). Experiments were always done in triplicate. Data is presented as a percentage of proliferation of untreated cells for each respective time point.

### DNA fragmentation assay analyzed by flow cytometry

The 3′OH termini in DNA breaks were measured by attaching fluorescent tagged deoxyuridine triphosphate nucleotides FITC-dUTP, in a reaction catalyzed by terminal deoxynucleotidyl transferase (TdT) using the Apo-Direct™ Kit (Phoenix Flow Systems, San Diego, CA, USA) purchased from Pharmingen, San Diego, CA. The amount of incorporated fluorescein was detected by flow cytometry.

Cell lines ($1 \times 10^6$ in T-25 flasks) were incubated with anti-Her-2/neu antibody (final concentrations: 0.025 µg/ml, 0.25 µg/ml) for 24, 48, 72 and 96 h, respectively. Untreated and treated cells were harvested, washed twice in phosphate buffered saline (PBS), fixed in 1% (w/v) paraformaldehyde in PBS (pH 7.2), for 15 min on ice. After sedimentation and two more washing steps, cells were resuspended in ice-cold 70% (v/v) ethanol and stored at –20˚C until further use (maximum: 3 weeks). According to the manufacturer's instructions, cells were washed twice in wash buffer, resuspended in 50 µl staining solution (10 µl reaction buffer, 0.75 µl TdT, 8 µl FITC-dUTP and 32 µl distilled water) and incubated for 1 h at 37˚C. Afterwards, 1 ml rinsing buffer was added. Cells were centrifuged ($1000 \times \boldsymbol{g}$) and rinsed again. Subsequently, cells were resuspended in one ml PI/RNAse solution and incubated in the dark at room temperature for 30 min. Subsequently, cell samples were analysed by flow cytometry on a FACScan (Becton Dickinson, CA, USA).

### Morphological evaluation of apoptosis

Cell morphology was performed by staining with May-Gruenwald-Giemsa. For this reason cells were cultured in chamber slides ('Lab-Tek Chamber Slide w/cover Glass Slide 2 Well') (Nalge Nunc International, Naperville, USA). Treated and untreated cells were stained with May-Gruenwald (Merck, Darmstadt, Germany) for 5 min. Subsequently cells were washed with distilled water (Leopold Pharma, Graz, Austria) for 5 min and stained with May-Gruenwald-Giemsa (Merck, Darmstadt, Germany; diluted 1:10) for 20 min. After a final wash with distilled water, samples were viewed under light microscopy. Cells were considered to be apoptotic according to the criteria introduced by Kerr et al (1972) which included: compaction of the nuclear chromatin, fragmentation of nuclei, condensation of the cytoplasm and separation of the cell into apoptotic bodies.

### Detection of bcl-2 by immunofluorescence

Cells ($1 \times 10^6$ in T-25 flasks) were incubated with anti-Her-2/neu antibody (final concentrations: 0.025 µg/ml, 0.25 µg/ml and 2.5 µg/ml) for 24, 48, 72 and 96 h, respectively. After harvesting and 3 washes with PBS cells were fixed and permeabilized with the fix & perm cell permeabilization kit (An der Grub, Bio Research Gmbh, Kaumberg, Austria). According to the manufacturer's instructions, $10^6$ cells/sample were resuspended in 100 µl fixation medium and incubated for 15 min at room temperature. Afterwards, 5 ml PBS were added. Cells were centrifuged (5 min at 300 $\boldsymbol{g}$) and subsequently resuspended in 100 µl permeabilization medium and 10 µl FITC-conjugated monoclonal mouse $IgG_1$ anti-human bcl-2 antibody (clone 124; DAKO, Glostrup, Denmark), vortexed at low speed for 2 s and incubated for 15 min at room temperature. After one more washing step, cells were analyzed by flow cytometry on a FACScan (Becton Dickinson, CA, USA).

### RESULTS

### Her-2/neu protein expression

As assessed by FACS analysis using a FITC-conjugated anti-Her-2/neu monoclonal antibody preparation, Her-2/neu expression ranged from 83–100% in cell lines SK-BR-3, HTB-24, HTB-25, HTB-27, HTB-128, HTB-130 and HTB-131, whereas no Her-2/neu protein was found on HTB-132 and HBL-100 cell lines (data not shown).

### p53 Genotype (Table 1) and protein expression p53 genotype was analysed by sequence analysis in eight breast cancer cell lines and one human mammary epithelial cell line. As shown in Table 1, wild type p53 was present in cell lines HTB-25, HTB-27, HTB-131 and HBL-100, whereas mutations in the p53

© 2001 Cancer Research Campaign

**Table 1** p53 gene mutations characterised in human breast cancer cell lines

| Cell line | Base change | EXON/CODON | Amino acid change |
|---|---|---|---|
| SK-BR-3 | CGC to CAC | 5/175 | Arg-His |
| HTB-24 | 26 bp del | 4/87–96 | frameshift |
| HTB-128 | TAC TGC | 7/236 | Tyr-Cys |
| HTB-130 | 7 bp Ins | 6/204 | frameshift |
| HTB-132 | CGT to CAT | 8/273 | Arg-His |
| HTB-131 | wild type | | |
| HTB-27 | wild type | | |
| HTB-25 | wild type | | |
| HBL-100 (human mammary epithelial) | wild type | | |

gene were found in SK-BR-3, HTB-24, HTB-128, HTB-130 and HTB-132 cells. Immunohistochemistry for p53 revealed intense nuclear staining in SK-BR-3 (Figure 1), HTB-128 and HTB-132 cells, whereas HTB-25 (Figure 2), HTB-27, HTB-131, HBL-100, HTB-24 and HTB-130 cell lines showed no nuclear staining. Thus, the p53 mutation of HTB-24 and HTB-130 cells was probably associated with loss of protein staining.

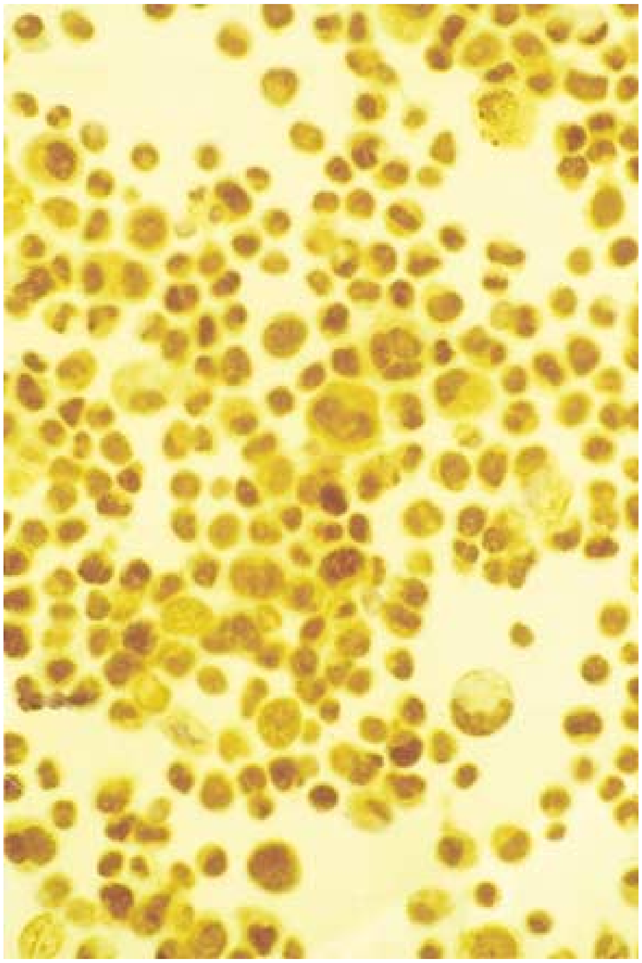

**Figure 1** Immunohistochemistry of SK-BR-3 cells for p53 protein showed intense nuclear staining

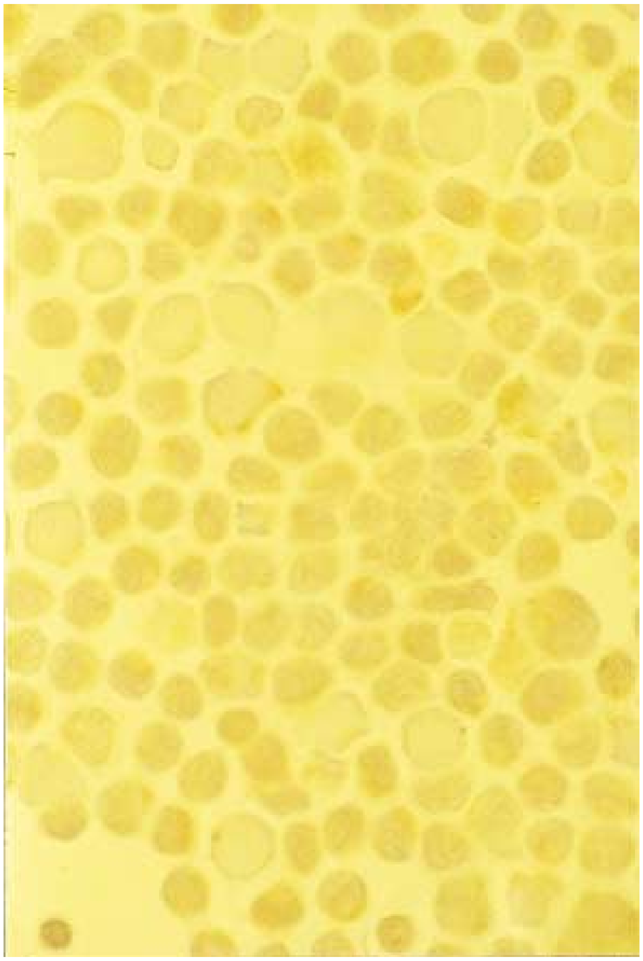

**Figure 2** Immunohistochemistry of HTB-25 cells for p53 protein showed no overexpression of p53

### Inhibition of cell proliferation by anti-Her-2/neu antibody (Figure 3)

As shown in Figure 3, the monoclonal anti-Her-2/neu antibody dose- and time-dependently inhibited the growth of Her-2/neu-positive cells, whereas the Her-2/neu-negative cell lines HTB-132 and HBL-100 remained unhindered. The maximum inhibition of cell proliferation was obtained with a final antibody concentration of 2.5 μg/ml after 96 h of incubation, as compared to untreated cells. Control experiments using non-specific mouse IgG1 antibody did not show any influence upon cell proliferation (data not shown).

### Induction of apoptosis: DNA fragmentation, analysis of cell-cycle position and DNA content (Table 2)

In summary, anti-Her-2/neu antibody was able to induce apoptosis dose-dependently in all HER-2/neu positive breast cancer cell lines irrespective of their p53 status with an optimal duration of incubation with 0.25 μg/ml anti-Her-2/neu antibody for 96 h. Thus, anti-Her-2/neu antibody-induced apoptosis was seen in similar degrees in cell lines with wild type p53 as well as in those with p53 mutations. No apoptosis was found in Her-2/neu negative cell lines HTB-132 and HBL-100 during an incubation period of up to 96 h. Control experiments with non-specific mouse $IgG_1$ antibody did not induce apoptosis within the panel of tested cell lines (data not shown).

*© 2001 Cancer Research Campaign*

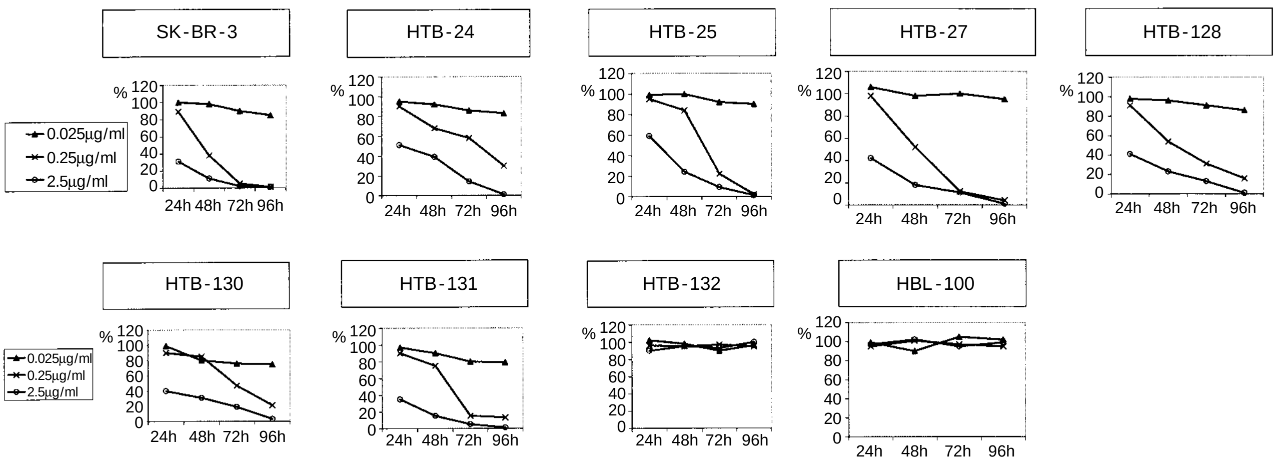

**Figure 3** Proliferation of SK-BR-3, HTB-24, HTB-25, HTB-27, HTB-128, HTB-130, HTB-131 and HTB-132 (all breast cancer) and HBL-100 (human mammary epithelial) cells during incubation with anti-HER-2/neu antibody in varying concentrations (0.025 µg/ml, 0.25 µg/ml, 2.5 µg/ml) for 24, 48, 72 and 96 h ($^3$H-thymidine incorporation assay). Data are presented as relative counts per minute of untreated cells (100%) for each respective time point

**Table 2** Percentage of apoptotic breast cancer cells after incubation with anti-Her-2/neu antibody (0.25 µg/ml) for 96 h

| Cell line | % apoptotic cells |
|---|---|
| SK-BR-3 | 97 |
| HTB-24 | 53 |
| HTB-25 | 95 |
| HTB-27 | 95 |
| HTB-128 | 76 |
| HTB-130 | 68 |
| HTB-131 | 83 |
| HTB-132 | 0 |
| HBL-100 (human mammary epithelial) | 0 |

In order to analyse cell cycle position, global DNA content was measured with PI counterstaining: treatment of Her-2/neu positive cell lines with anti-Her-2/neu antibody was associated with some G1-S arrest. In detail, the percentages of apoptosic cells after incubation with 0.25 µg/ml anti-Her-2/neu antibody for 96 h in correlation with cell cycle positions were 97% (G1: 72%; G2 & S: 25%) for SK-BR-3, 53% (G1: 40%; G2 & S: 13%) for HTB-24, 95% for HTB-25 (G1: 81%; G2 & S: 14%), 95% (G1: 64%; G2 & S: 31%) for HTB-27, 76% (G1: 48%; G2 & S: 28%) for HTB-128, 68% (G1: 57%; G2 & S: 11%) for HTB-130 and 83% (G1: 50%; G2 & S: 33%) for HTB-131 cells, respectively. No apoptosis was measured in HTB-132 and HBL-100 cells.

The lower concentration of anti-Her-2/neu antibody (0.025 µg/ml) was unable to induce apoptosis in either cell line after any length of incubation (24–96 h).

In addition, morphologic evaluation of cells shown to undergo apoptosis by flow cytometry was carried out. Within this context morphology of respective cells exhibited typical apoptotic features such as nuclear-chromatin compaction, cytoplasma condensation around the nucleus, cell shrinkage and apoptotic 'bodies' (data not shown).

### bcl-2 Protein expression

All nine native cell lines weakly expressed bcl-2 protein (10–20%). Treatment of these cell lines with anti-Her-2/neu antibody (final concentrations: 0.025 µg/ml, 0.25 µg/ml) for 24, 48, 72 and 96 h, respectively, did not modify the expression of bcl-2 protein (data not shown). However, in order to assess the influence of anti-Her-2/neu antibody on bcl-2 expression appropriately, inclusion of cell lines with variable bcl-2 expression would probably provide more accurate information in this regard.

### DISCUSSION

In the present paper, we report on the ability of anti-Her-2/neu antibody to induce apoptosis and inhibit proliferation of various Her-2/neu protein overexpressing breast cancer cell lines. In contrast, Her-2/neu negative control cell lines were not influenced either in their proliferative ability nor did they become apoptotic following exposure to anti-Her-2/neu antibody. These observations corroborate previous observations by other investigators (Harwerth et al, 1992, 1993; Brodowicz et al, 1997). In an attempt to further analyse the underlying molecular pattern resulting in these findings, the sequence of p53 was analysed and put into relation with the above results building upon previous insights on wild type p53 representing a regulator of appropriate inhibition of proliferation and induction of apoptosis following DNA damage (Brown and Wouters, 1998). It was surprising to find that anti-Her-2/neu antibody induced both, proliferation inhibition and apoptosis independently from p53 status. Thus, incubation of cell lines with anti-Her-2/neu antibody resulted in proliferation inhibition and apoptosis in a similar degree in all cell lines, and was solely dependent from Her-2/neu overexpression, but not from the presence of wild type p53. Although p53-mutated SK-BR-3, HTB-128 and HTB-132 cells showed intensive nuclear staining by immunohistochemistry, the p53 mutated HTB-24 and HTB-130 cells showed no nuclear staining. The p53 mutation in HTB-24 and HTB-130 cell lines was probably related to a loss of protein staining (Aas et al, 1996) due to a miss of the antibody binding site of the mutated protein or a complete absence of the protein (Thor et al, 1992).

p53 Has been shown to either induce inhibition of proliferation or apoptosis in the case of DNA damage and does so in collaboration with other regulators of cellular homeostasis including bax (Zha et al, 1997), bcl-2 (Reed, 1994; Kroemer, 1997) as well as p21 (Xiong et al, 1993; Harris, 1996), p27 (Harris, 1996) and cyclin E (Harris, 1996). It is interesting to note that bcl-2 which

 © 2001 Cancer Research Campaign acts as an inhibitor of apoptosis was obviously not involved in the generation of the current results, as all native cell lines expressed bcl-2 protein at very low levels. The fact that anti-Her-2/neu antibody exerted the biologic activity described above in a p53-independent manner puts it into line not only with cytotoxic agents including paclitaxel and vinca alkaloids, but also with death ligands such as tumour necrosis factor, Fas ligand and Apo2L which all have been shown to act independently from intact p53 in their ability to induce apoptosis (reviewed in Reed, 1999). Thus, the only variable obviously responsible for the efficacy of anti-Her-2/neu antibody in the present model was the fact of overexpression of Her-2/neu protein on target cells. This is in accordance with previous data obtained both in vitro (Harwerth et al, 1992) and in vivo (Harwerth et al, 1993) elaborating on the immunologic background of the activity of the agent and its necessity to identify an appropriate target for the initiation of its function.

Our data lend support to observations made in vivo on the efficacy of combined chemotherapeutic treatment with anti-Her-2/neu antibody in patients with breast cancer overexpressing Her-2/neu protein by alluding to the possibility of additive efficacy in targeting possibly various malignant cell populations by the combined approach. It is interesting to note in the present context that clinical results with paclitaxel have been substantially improved by the addition of anti-Her-2/neu antibody (Slamon et al, 1998, 2001; Norton et al, 1999) and both agents obviously act in a similar, p53-independent manner to achieve apoptosis of malignant cells (Wahl et al, 1996). Furthermore anti-Her-2/neu antibody was administered in combination with cisplatin (Pegram et al, 1998), adriamycin (Slamon et al, 2001) and navelbine (Burstein et al, 2001) in metastatic breast cancer patients. In contrast to paclitaxel, these drugs have been shown to act in a p53-dependent manner: sensitivity of several different cell lines to cisplatin correlated with wild-type p53 (O'Connor et al, 1997). In patients with advanced breast cancer clinical response to neoadjuvant anthracycline-containing chemotherapy was found to be dependent on normal p53 status in the respective breast tumors (Kandioler-Eckersberger et al, 2000). Thus, anthracyclines seem to trigger p53-dependent apoptosis within this context (Kandioler-Eckersberger et al, 2000). In the presence of mutant p53, a decreased sensitivity of cell lines to vinca alkaloids has been described (Zhang et al, 1998). However, with respect to antimitotic activity of vinca alkaloids, a p53 independent impact upon various cell lines was also described (O'Connor et al, 1997).

We conclude that anti-Her-2/neu antibody constitutes a valuable tool for the induction of p53-independent apoptosis in Her-2/neu overexpressing cells and might therefore, represent an important therapeutic modality for the translation of the present in vitro findings into the clinical setting.

### ACKNOWLEDGEMENT

The authors gratefully acknowledge expert technical assistance by Ms. Waclawa Kalinowski.

### REFERENCES

Aas T, Borresen AL, Geisler S, Smith-Sorensen B, Johnson H, Varhaug JE, Akslen LA and Lonning PE (1996) Specific p53 mutations are associated with de novo resistance to doxorubicin in breast cancer patients. *Nat Med* **2**: 811–814

Beham A, Marin MC, Fernandez A, Herrmann J, Brisbay S, Tari AM, Lopez-Berestein G, Lozano G, Sarkiss M and McDonnell TJ (1997) Bcl-2 inhibits p53 nuclear import following DNA damage. *Oncogene* **15**: 2767–2772

Bergh J, Norberg T, Sjogren S, Lindgren A and Holmberg L (1995) Complete sequencing of the p53 gene provides prognostic information in breast cancer patients, particularly in relation to adjuvant systemic therapy and radiotherapy. *Nat Med* **1**: 1029–1034

Bracey TS, Miller JC, Preece A and Paraskeva C (1995) γ-radiation-induced apoptosis in human colorectal adenoma and carcinoma cell lines can occur in the absence of wild type p53. *Oncogene* **10**: 2391–2396

Brodowicz T, Wiltschke C, Budinsky AC, Krainer M, Steger GG and Zielinski CC (1997) Soluble HER-2/neu neutralizes biologic effects of anti-HER-2/neu antibody on breast cancer cells in vitro. *Int J Cancer* **73**: 875–879

Brodowicz T, Wiltschke C, Kandioler-Eckersberger D, Grunt TW, Rudas M, Schneider SM, Hejna M, Budinsky A and Zielinski CC (1999) Inhibition of proliferation and induction of apoptosis in soft tissue sarcoma cells by interferon-alpha and retinoids. *Br J Cancer* **80**: 1350–1358

Brown JM and Wouters BG (1998) Apoptosis, p53, and tumor cell sensitivity to anticancer agents. *Cancer Res* **59**: 1391–1399

Buckbinder L, Talbott R, Velasco-Miguel S, Takenaka I, Faha B, Seizinger BR and Kley N (1995) Induction of the growth inhibitor IGF-binding protein 3 by p53. *Nature* **377**: 646–649

Burstein HJ, Kuter I, Campos SM, Gelman RS, Tribou L, Parker LM, Manola J, Younger J, Matulonis U, Bunnell CA, Patridge AH, Richardson PG, Clarke K, Shulman LN and Winer EP (2001) Clinical activity of trastuzumab and vinorelbine in women with HER2-overexpressing metastatic breast cancer. *J Clin Oncol* **19**: 2722–2730

Chiou SK, Rao L and White E (1994) Bcl-2 blocks p53-dependent apoptosis. *Mol Cell Biol* **14**: 2556–2563

Cobleigh MA, Vogel CL, Tripathy D, Robert NJ, Scholl S, Fehrenbacher L, Paton V, Shak S, Lieberman G and Slamon DJ (1998) Efficacy and safety of herceptin™ (humanized anti-Her2 antibody) as a single agent in 222 women with Her2 overexpression who relapsed following chemotherapy for metastatic breast cancer. *Proc Am Soc Clin Oncol* **17**: 376a (Abstr.)

Cobleigh MA, Vogel CL, Tripathy D, Robert NJ, Scholl S, Fehrenbacher L, Wolter JM, Paton V, Shak S, Lieberman G and Slamon DJ (1999) Multinational study of the efficacy and safety of humanized anti-HER2 monoclonal antibody in women who have HER2-overexpressing metastatic breast cancer that has progressed after chemotherapy for metastatic disease. *J Clin Oncol* **17**: 2639–2648

Coussens L, Yang-Feng TL, Liao YC, Chen E, Gray A, McGrath J, Seeburg PH, Libermann TA, Schlessinger J and Francke U (1985) Tyrosine kinase receptor with extensive homology to EGF receptor shares chromosomal location with *neu* oncogene. *Science* **230**: 1132–1139

Delia D, Aiello A, Lombardi L, Pelicci PG, Grignani Fr, Grignani Fa, Formelli F, Menard S, Costa A, Veronesi U and Pierotti MA (1993) N-(4-hydroxyphenyl)retinamide induces apoptosis of malignant hemopoietic cell lines including those unresponsive to retinoic acid. *Cancer Res* **53**: 6036–6041

Di Leonardo A, Linke SP, Clarkin K and Wahl GM (1994) DNA damage triggers a prolonged p53-dependent G1 arrest and long-term induction of Cipl in normal human fibroblasts. *Genes Dev* **8**: 2540–2551

Dragovich T, Rudin CM and Thompson CB (1998) Signal transduction pathways that regulate cell survival and cell death. *Oncogene* **17**: 3207–3213

Dyson N (1998) The regulation of E2F by pRB-family proteins. *Genes Dev* **12**: 2245–2262

Elledge RM, Gray R, Mansour E, Yu Y, Clark GM and Ravdin P (1995) Accumulation of p53 protein as a possible predictor of response to adjuvant combination chemotherapy with cyclophosphamide, methotrexate, fluorouracil, and prednisone for breast cancer. *J Natl Cancer Inst* **87**: 1254–1256

Fornier M, Seidman AD, Esteva FJ, Theodoulou M, Moynahan M, Currie V, Moasser M, Sklarin N, Gilewski T, Surbone A, Denton C, Bacotti D, Willey J, Bach A, Reuter V, Hortobagyi G, Norton L and Hudis C (1999) Weekly herceptin + 1 hour taxol: phase II study in Her2 overexpressing and non-overexpressing metastatic breast cancer. *Proc Am Soc Clin Oncol* **18**: 482a (Abstr.)

Harris CC (1996) Structure and function of the p53 tumor suppressor gene: clues for rational cancer therapeutic strategies. *J Natl Cancer Inst* **88**: 1442–1455

Harvey M, Sands AT, Weiss RS, Hegi ME, Wiseman RW, Pantazis P, Giovanella BC, Tainsky MA, Bradley A and Donehower LA (1993) In vitro growth characteristics of embryo fibroblasts isolated from p53-deficient mice. *Oncogene* **8**: 2457–2467

Harwerth IM, Wels W, Marte BM and Hynes NE (1992) Monoclonal antibodies against the extracellular domain of the erbB-2 receptor function as partial ligand agonists. *J Biol Chem* **267**: 15160–15167

Harwerth IM, Wels W, Schlegel J, Muller M and Hynes NE (1993) Monoclonal antibodies directed to the erbB-2 receptor inhibit in vivo tumour cell growth. *Br J Cancer* **68**: 1140–1145

© 2001 Cancer Research Campaign 

Hockenbery DM, Oltvai ZN, Yin XM, Milliman CL and Korsmeyer SJ (1993) Bcl-2 functions in an antioxidant pathway to prevent apoptosis. *Cell* **75**: 241–251

Israeli D, Tessler E, Haupt Y, Elkeles A, Wilder S, Amson R, Telerman A and Oren M (1997) A novel p53-inducible gene, PAG608, encodes a nuclear zinc finger protein whose overexpression promotes apoptosis. *EMBO J* **16**: 4384–4392

Johnson TM, Yu ZX, Ferrans VJ, Lowenstein RA and Finkel T (1996) Reactive oxygen species are downstream mediators of p53-dependent apoptosis. *Proc Natl Acad Sci USA* **93**: 11848–11852

Kandioler-Eckersberger D, Ludwig C, Rudas M, Kappel S, Janschek E, Wenzel C, Schlagbauer-Wadl H, Mittlböck M, Gnant M, Steger G and Jakesz R (2000) TP53 mutation and p53 overexpression for prediction of response to neoadjuvant treatment in breast cancer patients. *Clin Cancer Res* **6**: 50–56

Kastan MB, Onyekwere O, Sidransky D, Vogelstein B and Craig RW (1991) Participation of p53 in the cellular response to DNA damage. *Cancer Res* **51**: 6304–6311

Kerbel RS (1999) Some recent advances in preclinical aspects of treating cancer by inhibition of tumor angiogenesis. In *ASCO Educational Book*, pp 4–7

Kerr JFR, Wyllie AH and Currie AR (1972) Apoptosis: a basic biological phenomenon with wide ranging implications in tissue kinetics. *Br J Cancer* **26**: 239–257

Kraus MH, Popescu NC, Amsbaugh SC and King CR (1987) Overexpression of the EGF receptor-related proto-oncogene erbB-2 in human mammary tumor cell lines by different molecular mechanisms. *EMBO J* **6**: 605–610

Kroemer G (1997) The proto-oncogene bcl-2 and its role in regulating apoptosis. *Nature Med* **3**: 614–620

Lehman TA, Bennett WP, Metcalf RA, Welsh JA, Ecker J, Modali RV, Ullrich S, Romano JW, Appella E, Testa JR, Gerwin BI and Harris CC (1991) P53 mutations, ras mutations, and p53-heat shock 70 protein complexes in human lung carcinoma cell lines. *Cancer Res* **51**: 4090–4096

Lupu R, Colomer R, Zugmaier G, Sarup J, Shepard M, Slamon D and Lippman ME (1990) Direct interaction of a ligand for the erbB2 oncogene product with the EGF receptor and $p185^{erbB2}$. *Science* **249**: 1552–1555

Naumovski L and Cleary ML (1996) The p53-binding protein 53BP2 also interacts with bcl2 and impedes cell cycle progression at G2/M. *Mol Cell Biol* **16**: 3884–3892

Norton L, Slamon D, Leyland-Jones B, Wolter J, Fleming T, Eiermann W, Baselga J, Mendelsohn J, Bajamonde A, Ash M and Shak S (1999) Overall survival advantage to simultaneous chemotherapy plus the humanized anti-her2 monoclonal antibody herceptin in Her2-overexpressing metastatic breast cancer. *Proc Am Soc Clin Oncol* **18**: 483a (Abstr.)

O'Connor PM, Jackman J, Bae I, Myers TG, Fan S, Mutoh M, Scudiero DA, Monks A, Sausville EA, Weinstein JN, Friend S, Fornace AJ and Kohn KW (1997) Characterization of the p53 tumor suppressor pathway in cell lines of the National Cancer Institute anticancer drug screen and correlations with the growth-inhibitory potency of 123 anticancer agents. *Cancer Res* **57**: 4285–4300

Owen-Schaub LB, Zhang W, Cusack JC, Angelo LS, Santee SM, Fujiwara T, Roth JA, Deisseroth AB, Zhang WW, Kruzel E and Radinsky R (1995) Wild-type human p53 and a temperature-sensitive mutant induce Fas/APO-1 expression. *Mol Cell Biol* **15**: 3032–3040

Pegram MD, Lipton A, Hayes DF, Weber BL, Baselga JM, Tripathy D, Baly D, Baughman SA, Twaddell T, Glaspy JA and Slamon DJ (1998) Phase II study of receptor-enhanced chemosensitivity using recombinant humanized anti-p185HER2/neu monoclonal antibody plus cisplatin in patients with HER2/neu-overexpressing metastatic breast cancer refractory to chemotherapy treatment. *J Clin Oncol* **16**: 2659–2671

Reed JC (1994) Bcl-2 and the regulation of programmed cell death. *J Cell Biol* **124**: 1–6

Reed JC (1999) Dysregulation of apoptosis in cancer. *J Clin Oncol* **17**: 2941–2953

Sarkis AS, Bajorin DF, Reuter VE, Herr HW, Netto G, Zhang ZF, Schultz PK, Cordon-Cardo C and Scher HI (1995) Prognostic value of p53 nuclear overexpression in patients with invasive bladder cancer treated with neoadjuvant MVAC. *J Clin Oncol* **13**: 1384–1390

Shao ZM, Dawson MI, Li XS, Rishi AK, Sheikh MS, Han QX, Ordonez JV, Shroot B and Fontana JA (1995) p53 independent $G_0G_1$ arrest and apoptosis induced by a novel retinoid in human breast cancer cells. *Oncogene* **11**: 493–504

Sionov RV and Haupt Y (1999) The cellular response to p53: the decision between life and death. *Oncogene* **18**: 6145–6157

Slamon D, Leyland-Jones B, Shak S, Paton V, Bajamonde A, Fleming T, Eiermann W, Wolter J, Baselga J and Norton L (1998) Addition of herceptin™ to first line chemotherapy for Her2 overexpressing metastatic breast cancer markedly increases anticancer activity: a randomized, multinational controlled phase III trial. *Proc Am Soc Clin Oncol* **17**: 377a (Abstr.)

Slamon DJ, Leyland-Jones B, Shak S, Fuchs H, Paton V, Bajamonde A, Fleming T, Eiermann W, Wolter J, Pegram M, Baselga J and Norton L (2001) Use of chemotherapy plus a monoclonal antibody against HER2 for metastatic breast cancer that overexpress HER2. *N Engl J Med* **344**: 783–792

Susin SA, Lorenzo HK, Zamzami N, Marzo I, Snow BE, Brothers GM, Mangion J, Jacotot E, Constantini P, Loeffler M, Larochette N, Goodlett DR, Aebersold R, Siderovski DP, Penninger JM and Kroemer G (1999) Molecular characterization of mitochondrial apoptosis-inducing factor. *Nature* **397**: 441–446

Thompson CB (1995) Apoptosis in the pathogenesis and treatment of disease. *Science* **267**: 1456–1462

Thor AD, Moore DH, Edgerton SM, Kawasaki ES, Reihsaus E, Lynch HT, Marcus JN, Schwartz L, Chen LC, Mayall BH and Smith HS (1992) Accumulation of p53 tumor suppressor gene protein: an independent marker of prognosis in breast cancers. *J Natl Cancer Inst* **84**: 845–855

Venot C, Maratrat M, Dureuil C, Conseiller E, Bracco L and Debussche L (1998) The requirement for the p53 proline-rich functional domain for mediation of apoptosis is correlated with specific PIG3 gene transactivation and with transcriptional repression. *EMBO J* **17**: 4668–4679

Wahl AF, Donaldson KL, Fairchild C, Lee FY, Foster SA, Demers GW and Galloway DA (1996) Loss of normal p53 function confers to sensitization to Taxol by increasing G2/M arrest and apoptosis. *Na Med* **2**: 72–79

Wang XW, Vermeulen W, Coursen JD, Gibson M, Lupold SE, Forrester K, Xu G, Elmore L, Yeh H, Hoeijmakers JH and Harris CC (1996) The XPB and XPD DNA helicases are components of the p53-mediated apoptosis pathway. *Genes Dev* **10**: 1219–1232

Wu GS, Burns TF, McDonald ER 3rd, Meng RD, Kao G, Muschel R, Yen T and El Deiry WS (1999) Induction of the TRAIL receptor KILLER/DR5 in p53-dependent apoptosis but not growth arrest. *Oncogene* **18**: 6411–6418

Xiong Y, Hannon GJ, Zhang H, Casso D, Kobayashi R and Beach D (1993) p21 is a universal inhibitor of cyclin kinases. *Nature* **366**: 701–704

Yin C, Knudson CM, Korsmeyer SJ and Van Dyke T (1997) Bax suppresses tumorigenesis and stimulates apoptosis in vivo. *Nature* **385**: 637–640

Yonish-Rouach E, Resnitzky D, Lotem J, Sachs L, Kimchi A and Oren M (1991) Wild-type p53 induces apoptosis of myeloid leukaemic cells that is inhibited by interleukin-6. *Nature* **352**: 345–347

Zha H and Reed JC (1997) Heterodimerization-independent functions of cell death regulatory proteins bax and bcl-2 in yeast and mammalian cells. *J Biol Chem* **272**: 31482–31488

Zhang CC, Yang JM, White E, Murphy M, Levine A and Hait WN (1998) The role of MAP4 expression in the sensitivity to paclitaxel and resistance to vinca alkaloids in p53 mutant cells. *Oncogene* **16**: 1617–1624

*© 2001 Cancer Research Campaign*

## References

[PDF_00608] Aas T., Børresen A. L., Geisler S., Smith-Sørensen B., Johnsen H., Varhaug J. E., Akslen L. A., Lønning P. E. (1996). Specific P53 mutations are associated with de novo resistance to doxorubicin in breast cancer patients.. Nat Med.

[PDF_00612] Beham A., Marin M. C., Fernandez A., Herrmann J., Brisbay S., Tari A. M., Lopez-Berestein G., Lozano G., Sarkiss M., McDonnell T. J. (1997). Bcl-2 inhibits p53 nuclear import following DNA damage.. Oncogene.

[PDF_00614] Bergh J., Norberg T., Sjögren S., Lindgren A., Holmberg L. (1995). Complete sequencing of the p53 gene provides prognostic information in breast cancer patients, particularly in relation to adjuvant systemic therapy and radiotherapy.. Nat Med.

[PDF_00618] Bracey T. S., Miller J. C., Preece A., Paraskeva C. (1995). Gamma-radiation-induced apoptosis in human colorectal adenoma and carcinoma cell lines can occur in the absence of wild type p53.. Oncogene.

[PDF_00621] Brodowicz T., Wiltschke C., Budinsky A. C., Krainer M., Steger G. G., Zielinski C. C. (1997). Soluble HER-2/neu neutralizes biologic effects of anti-HER-2/neu antibody on breast cancer cells in vitro.. Int J Cancer.

[PDF_00624] Brodowicz T., Wiltschke C., Kandioler-Eckersberger D., Grunt T. W., Rudas M., Schneider S. M., Hejna M., Budinsky A., Zielinski C. C. (1999). Inhibition of proliferation and induction of apoptosis in soft tissue sarcoma cells by interferon-alpha and retinoids.. Br J Cancer.

[PDF_00628] Brown J. M., Wouters B. G. (1999). Apoptosis, p53, and tumor cell sensitivity to anticancer agents.. Cancer Res.

[PDF_00630] Buckbinder L., Talbott R., Velasco-Miguel S., Takenaka I., Faha B., Seizinger B. R., Kley N. (1995). Induction of the growth inhibitor IGF-binding protein 3 by p53.. Nature.

[PDF_00633] Burstein H. J., Kuter I., Campos S. M., Gelman R. S., Tribou L., Parker L. M., Manola J., Younger J., Matulonis U., Bunnell C. A. (2001). Clinical activity of trastuzumab and vinorelbine in women with HER2-overexpressing metastatic breast cancer.. J Clin Oncol.

[PDF_00638] Chiou S. K., Rao L., White E. (1994). Bcl-2 blocks p53-dependent apoptosis.. Mol Cell Biol.

[PDF_00645] Cobleigh M. A., Vogel C. L., Tripathy D., Robert N. J., Scholl S., Fehrenbacher L., Wolter J. M., Paton V., Shak S., Lieberman G. (1999). Multinational study of the efficacy and safety of humanized anti-HER2 monoclonal antibody in women who have HER2-overexpressing metastatic breast cancer that has progressed after chemotherapy for metastatic disease.. J Clin Oncol.

[PDF_00651] Coussens L., Yang-Feng T. L., Liao Y. C., Chen E., Gray A., McGrath J., Seeburg P. H., Libermann T. A., Schlessinger J., Francke U. (1985). Tyrosine kinase receptor with extensive homology to EGF receptor shares chromosomal location with neu oncogene.. Science.

[PDF_00656] Delia D., Aiello A., Lombardi L., Pelicci P. G., Grignani F., Grignani F., Formelli F., Menard S., Costa A., Veronesi U. (1993). N-(4-hydroxyphenyl)retinamide induces apoptosis of malignant hemopoietic cell lines including those unresponsive to retinoic acid.. Cancer Res.

[PDF_00659] Di Leonardo A., Linke S. P., Clarkin K., Wahl G. M. (1994). DNA damage triggers a prolonged p53-dependent G1 arrest and long-term induction of Cip1 in normal human fibroblasts.. Genes Dev.

[PDF_00662] Dragovich T., Rudin C. M., Thompson C. B. (1998). Signal transduction pathways that regulate cell survival and cell death.. Oncogene.

[PDF_00664] Dyson N. (1998). The regulation of E2F by pRB-family proteins.. Genes Dev.

[PDF_00666] Elledge R. M., Gray R., Mansour E., Yu Y., Clark G. M., Ravdin P., Osborne C. K., Gilchrist K., Davidson N. E., Robert N. (1995). Accumulation of p53 protein as a possible predictor of response to adjuvant combination chemotherapy with cyclophosphamide, methotrexate, fluorouracil, and prednisone for breast cancer.. J Natl Cancer Inst.

[PDF_00676] Harris C. C. (1996). Structure and function of the p53 tumor suppressor gene: clues for rational cancer therapeutic strategies.. J Natl Cancer Inst.

[PDF_00678] Harvey M., Sands A. T., Weiss R. S., Hegi M. E., Wiseman R. W., Pantazis P., Giovanella B. C., Tainsky M. A., Bradley A., Donehower L. A. (1993). In vitro growth characteristics of embryo fibroblasts isolated from p53-deficient mice.. Oncogene.

[PDF_00682] Harwerth I. M., Wels W., Marte B. M., Hynes N. E. (1992). Monoclonal antibodies against the extracellular domain of the erbB-2 receptor function as partial ligand agonists.. J Biol Chem.

[PDF_00685] Harwerth I. M., Wels W., Schlegel J., Müller M., Hynes N. E. (1993). Monoclonal antibodies directed to the erbB-2 receptor inhibit in vivo tumour cell growth.. Br J Cancer.

[PDF_00690] Hockenbery D. M., Oltvai Z. N., Yin X. M., Milliman C. L., Korsmeyer S. J. (1993). Bcl-2 functions in an antioxidant pathway to prevent apoptosis.. Cell.

[PDF_00692] Israeli D., Tessler E., Haupt Y., Elkeles A., Wilder S., Amson R., Telerman A., Oren M. (1997). A novel p53-inducible gene, PAG608, encodes a nuclear zinc finger protein whose overexpression promotes apoptosis.. EMBO J.

[PDF_00695] Johnson T. M., Yu Z. X., Ferrans V. J., Lowenstein R. A., Finkel T. (1996). Reactive oxygen species are downstream mediators of p53-dependent apoptosis.. Proc Natl Acad Sci U S A.

[PDF_00698] Kandioler-Eckersberger D., Ludwig C., Rudas M., Kappel S., Janschek E., Wenzel C., Schlagbauer-Wadl H., Mittlböck M., Gnant M., Steger G. (2000). TP53 mutation and p53 overexpression for prediction of response to neoadjuvant treatment in breast cancer patients.. Clin Cancer Res.

[PDF_00702] Kastan M. B., Onyekwere O., Sidransky D., Vogelstein B., Craig R. W. (1991). Participation of p53 protein in the cellular response to DNA damage.. Cancer Res.

[PDF_00707] Kerr J. F., Wyllie A. H., Currie A. R. (1972). Apoptosis: a basic biological phenomenon with wide-ranging implications in tissue kinetics.. Br J Cancer.

[PDF_00710] Kraus M. H., Popescu N. C., Amsbaugh S. C., King C. R. (1987). Overexpression of the EGF receptor-related proto-oncogene erbB-2 in human mammary tumor cell lines by different molecular mechanisms.. EMBO J.

[PDF_00713] Kroemer G. (1997). The proto-oncogene Bcl-2 and its role in regulating apoptosis.. Nat Med.

[PDF_00715] Lehman T. A., Bennett W. P., Metcalf R. A., Welsh J. A., Ecker J., Modali R. V., Ullrich S., Romano J. W., Appella E., Testa J. R. (1991). p53 mutations, ras mutations, and p53-heat shock 70 protein complexes in human lung carcinoma cell lines.. Cancer Res.

[PDF_00719] Lupu R., Colomer R., Zugmaier G., Sarup J., Shepard M., Slamon D., Lippman M. E. (1990). Direct interaction of a ligand for the erbB2 oncogene product with the EGF receptor and p185erbB2.. Science.

[PDF_00723] Naumovski L., Cleary M. L. (1996). The p53-binding protein 53BP2 also interacts with Bc12 and impedes cell cycle progression at G2/M.. Mol Cell Biol.

[PDF_00731] O'Connor P. M., Jackman J., Bae I., Myers T. G., Fan S., Mutoh M., Scudiero D. A., Monks A., Sausville E. A., Weinstein J. N. (1997). Characterization of the p53 tumor suppressor pathway in cell lines of the National Cancer Institute anticancer drug screen and correlations with the growth-inhibitory potency of 123 anticancer agents.. Cancer Res.

[PDF_00736] Owen-Schaub L. B., Zhang W., Cusack J. C., Angelo L. S., Santee S. M., Fujiwara T., Roth J. A., Deisseroth A. B., Zhang W. W., Kruzel E. (1995). Wild-type human p53 and a temperature-sensitive mutant induce Fas/APO-1 expression.. Mol Cell Biol.

[PDF_00740] Pegram M. D., Lipton A., Hayes D. F., Weber B. L., Baselga J. M., Tripathy D., Baly D., Baughman S. A., Twaddell T., Glaspy J. A. (1998). Phase II study of receptor-enhanced chemosensitivity using recombinant humanized anti-p185HER2/neu monoclonal antibody plus cisplatin in patients with HER2/neu-overexpressing metastatic breast cancer refractory to chemotherapy treatment.. J Clin Oncol.

[PDF_00746] Reed J. C. (1994). Bcl-2 and the regulation of programmed cell death.. J Cell Biol.

[PDF_00748] Reed J. C. (1999). Dysregulation of apoptosis in cancer.. J Clin Oncol.

[PDF_00749] Sarkis A. S., Bajorin D. F., Reuter V. E., Herr H. W., Netto G., Zhang Z. F., Schultz P. K., Cordon-Cardo C., Scher H. I. (1995). Prognostic value of p53 nuclear overexpression in patients with invasive bladder cancer treated with neoadjuvant MVAC.. J Clin Oncol.

[PDF_00753] Shao Z. M., Dawson M. I., Li X. S., Rishi A. K., Sheikh M. S., Han Q. X., Ordonez J. V., Shroot B., Fontana J. A. (1995). p53 independent G0/G1 arrest and apoptosis induced by a novel retinoid in human breast cancer cells.. Oncogene.

[PDF_00758] Sionov R. V., Haupt Y. (1999). The cellular response to p53: the decision between life and death.. Oncogene.

[PDF_00765] Slamon D. J., Leyland-Jones B., Shak S., Fuchs H., Paton V., Bajamonde A., Fleming T., Eiermann W., Wolter J., Pegram M. (2001). Use of chemotherapy plus a monoclonal antibody against HER2 for metastatic breast cancer that overexpresses HER2.. N Engl J Med.

[PDF_00769] Susin S. A., Lorenzo H. K., Zamzami N., Marzo I., Snow B. E., Brothers G. M., Mangion J., Jacotot E., Costantini P., Loeffler M. (1999). Molecular characterization of mitochondrial apoptosis-inducing factor.. Nature.

[PDF_00774] Thompson C. B. (1995). Apoptosis in the pathogenesis and treatment of disease.. Science.

[PDF_00776] Thor A. D., Moore DH I. I., Edgerton S. M., Kawasaki E. S., Reihsaus E., Lynch H. T., Marcus J. N., Schwartz L., Chen L. C., Mayall B. H. (1992). Accumulation of p53 tumor suppressor gene protein: an independent marker of prognosis in breast cancers.. J Natl Cancer Inst.

[PDF_00780] Venot C., Maratrat M., Dureuil C., Conseiller E., Bracco L., Debussche L. (1998). The requirement for the p53 proline-rich functional domain for mediation of apoptosis is correlated with specific PIG3 gene transactivation and with transcriptional repression.. EMBO J.

[PDF_00784] Wahl A. F., Donaldson K. L., Fairchild C., Lee F. Y., Foster S. A., Demers G. W., Galloway D. A. (1996). Loss of normal p53 function confers sensitization to Taxol by increasing G2/M arrest and apoptosis.. Nat Med.

[PDF_00787] Wang X. W., Vermeulen W., Coursen J. D., Gibson M., Lupold S. E., Forrester K., Xu G., Elmore L., Yeh H., Hoeijmakers J. H. (1996). The XPB and XPD DNA helicases are components of the p53-mediated apoptosis pathway.. Genes Dev.

[PDF_00792] Wu G. S., Burns T. F., McDonald E. R., Meng R. D., Kao G., Muschel R., Yen T., el-Deiry W. S. (1999). Induction of the TRAIL receptor KILLER/DR5 in p53-dependent apoptosis but not growth arrest.. Oncogene.

[PDF_00794] Xiong Y., Hannon G. J., Zhang H., Casso D., Kobayashi R., Beach D. (1993). p21 is a universal inhibitor of cyclin kinases.. Nature.

[PDF_00796] Yin C., Knudson C. M., Korsmeyer S. J., Van Dyke T. (1997). Bax suppresses tumorigenesis and stimulates apoptosis in vivo.. Nature.

[PDF_00798] Yonish-Rouach E., Resnitzky D., Lotem J., Sachs L., Kimchi A., Oren M. (1991). Wild-type p53 induces apoptosis of myeloid leukaemic cells that is inhibited by interleukin-6.. Nature.

[PDF_00801] Zha H., Reed J. C. (1997). Heterodimerization-independent functions of cell death regulatory proteins Bax and Bcl-2 in yeast and mammalian cells.. J Biol Chem.

[PDF_00804] Zhang C. C., Yang J. M., White E., Murphy M., Levine A., Hait W. N. (1998). The role of MAP4 expression in the sensitivity to paclitaxel and resistance to vinca alkaloids in p53 mutant cells.. Oncogene.

